# Pivotal influence of ligand field stabilization energy on the extraction order of divalent metal ions by acidic extractants

**DOI:** 10.1039/d6re00168h

**Published:** 2026-06-15

**Authors:** Stijn Raiguel, Koen Binnemans

**Affiliations:** a KU Leuven, Department of Chemistry Celestijnenlaan 200F, P.O. box 2404 B-3001 Leuven Belgium stijn.raiguel@kuleuven.be

## Abstract

Even after decades of industrial use, the fundamental factors governing the selectivity of acidic extractants for various divalent metal ions in the fourth period of the periodic system have not yet been identified. In this paper, the case is made for ligand field stabilization energy as the principal quantity determining the extractability of a divalent metal ion by a particular acidic extractant. This stabilizing energetic contribution results from non-isotropic (covalent) interactions between the metal ion and the extractant, which acts as an inner-sphere ligand in the metal–extractant complex. The extractability of a metal ion appears to be largely determined by the difference in ligand field stabilization energy in the organic *versus* the aqueous phase. In order to investigate this correlation, the magnitude of the splitting of the d-orbitals in the metal–extractant complexes was probed by ultraviolet–visible–near-infrared absorption spectroscopy. Crystal field theory was applied to estimate the anisotropic contribution to the stability of each metal–extractant combination (ligand field stabilization energy), and of the hydrated metal ion in the aqueous phase. It was found that the anisotropic contribution is diminished upon extraction by extractants that weakly split the d-orbitals. This leads to preferential extraction of metal ions in which the electron configuration results in zero covalent contribution. Conversely, the anisotropic contribution becomes more favorable upon extraction by extractants which induce a large splitting of the d-orbitals. Hence, these extractants preferentially extract metal ions with a large covalent contribution to the total energy balance. Extractants with an intermediate splitting of d-orbitals exhibit little difference in covalent stabilization between both phases, and hence the extraction sequence is primarily determined by electrostatic effects, governed by the charge density of the metal ion. The model was applied to phosphoric, phosphonic, phosphinic, dithiophosphinic and carboxylic acid extractants, as well as to β-diketone and β-hydroxyketoxime extractants.

## Abstract 2

A model for the selectivity of acidic extractants is proposed, based on a striking correlation between the absorption spectrum of the metal–extractant complex and the order of extraction of divalent metal ions.

## Graphical abstract

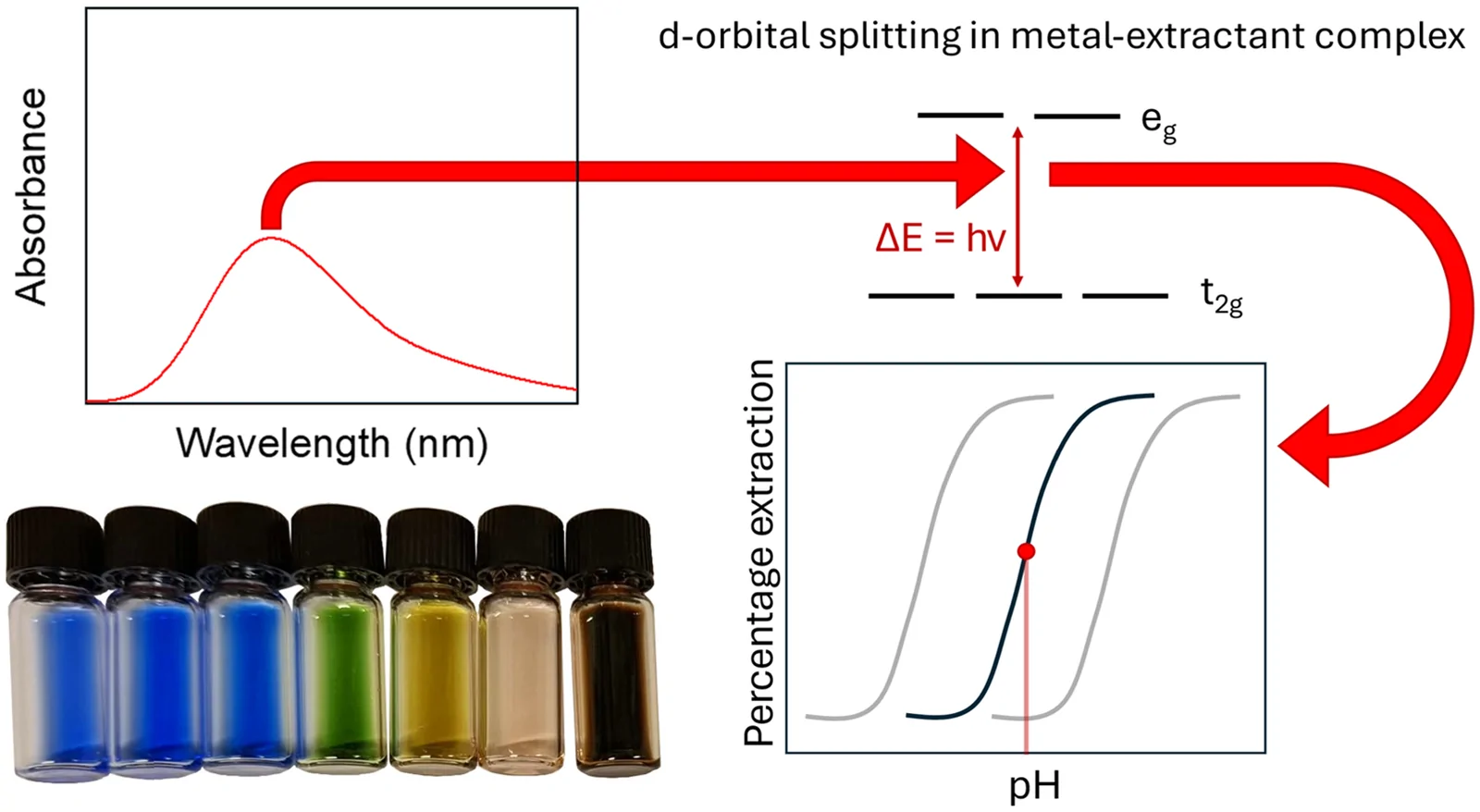

## Introduction

Solvent extraction (SX) has been a mainstay in hydrometallurgy for several decades, since its initial development for uranium purification in the early 1940s in the framework of the Manhattan project.^1^ The first acidic extractant to be introduced in an industrial setting was bis(2-ethylhexyl)phosphoric acid (D2EHPA) in 1955.^2^ An array of other acidic extractants have since entered the market, including phosphonic, phosphinic, dithiophosphinic and carboxylic acids, β-ketoximes and β-diketones. These acidic extractants all share the same mechanism of extraction, involving the interfacial exchange of cations (usually protons) for the desired metal ion in the aqueous phase (eqn (1)). A metal–extractant complex is thereby formed in the organic phase:1
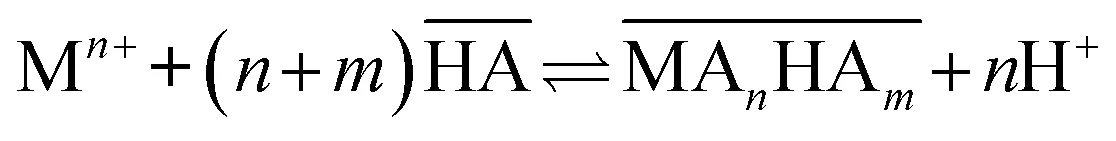
where M^*n*+^ represents an *n*-valent metal ion, A^−^ the conjugate base of the acidic extractant, and overbars denote species in the organic phase. Note that the metal–extractant complex may be oligomeric or polymeric at high loading for some extractants.^3,4^ As the reaction represented by eqn (1) entails the release of protons into the aqueous phase, extraction is a pH-dependent process. The pH at which extraction occurs is dependent on the nature of both the extractant and the extracted metal ion. This leads to an *order of extraction* for each extractant, arranging the metals from strongly extracted (*i.e.* extraction at low pH) to poorly extracted (extraction at high pH only).

In spite of the technological maturity of acidic extractants, the exact factors governing the relative extractability of metal ions have not been elucidated. For divalent transition metal ions in particular, it has become evident that every extractant is characterized by a specific sequence of metals, the origin of which is not immediately evident.^5,6^eqn (1) implies that the pH at which extraction occurs will be determined by both the acidity of the extractant and the stability of the metal–extractant complex. The former is inherent to the extractant and affects all *n*-valent metal ions equally. The latter will determine the order in which metal ions are extracted by the extractant, with the most strongly extracted metal ions forming the most stable complex. Supramolecular aggregation of the metal–extractant complex may also influence extraction.^4,7^

For divalent metal ions, the origin of the order of selectivity remains poorly understood. On the other hand, the trivalent rare-earth ions, which are dominated by electrostatic interactions, are usually extracted in order of decreasing ionic radius (*i.e.* increasing charge density).^8^ Charge density also explains the high affinity of acidic extractants for the trivalent Fe(iii) ion. This argument does not extend to the divalent fourth period ions, as they are not generally extracted in order of charge density. For Cyanex 301, the soft Lewis basicity of the sulfur donor atoms has been invoked in accounting for the selectivity of this extractant for certain soft metal ions, but this rationalization does not provide a satisfactory explanation for the behavior observed for the divalent fourth period metal ions, either.^9^ Some research has focused on the Co(ii)/Ni(ii) pair by organophosphorus acid extractants, because of its economic relevance.^7,10–13^ Unfortunately, the hypotheses proposed for this pair of metal ions (aggregation, steric hindrance and preferred coordination geometry) do not extend to other metal ions in the fourth period of the d-block, or even to other acidic extractants. Research on selectivity trends for other divalent metal ions has been sparse. A general, overarching model to account for the order of extraction of these ions remains lacking.

**Fig. 1:**
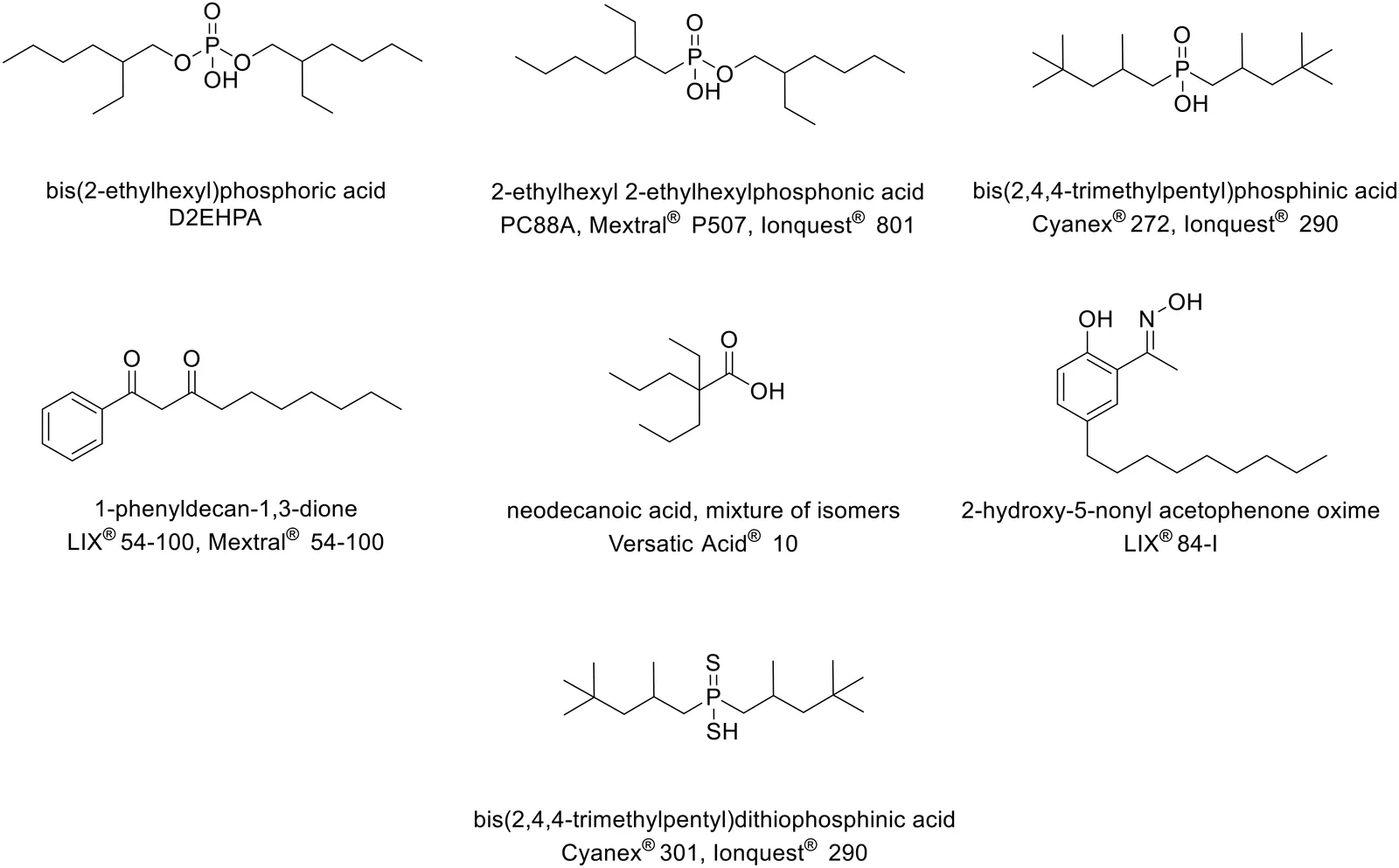
Structures of the extractants studied in this work, as well as brand names under which they are commercially available. Note that commercial production of Cyanex 301 and LIX 54-100 has ceased.

In this work, we present a general model to account for selectivity trends observed for divalent ions of the fourth period of the periodic system during extraction by acidic extractants, based on fundamental coordination chemistry principles. The theoretical basis of the model will first be elaborated, followed by an application of the model to a number of acidic extractants (Fig. 1). This application will rely on spectroscopic measurements to provide key parameters related to the electronic structure of the metal–extractant complexes.

It is worth noting that Mn and Co are sometimes encountered in in the trivalent state. For example, in ammoniacal media, Co(ii) is easily oxidized to Co(iii). While crystal field theory can also be separately applied to these trivalent ions, their high charge density will promote their extraction over divalent ions. Hence, these ions should be treated distinctly from their divalent form. Fe(ii) was excluded from the study, as oxidation to Fe(iii) by atmospheric oxygen is very rapid under the conditions applied in this work. Another noteworthy point is the fact that concentrated chloride systems may exhibit different selectivity, due to the formation of chloro complexes in the aqueous phase. This study pertains to sulfate solutions, when the hydrated ion can be assumed to exist exclusively in the aqueous phase.

## Experimental

### Chemicals and reagents

Sodium hydroxide (pearl, analytical reagent grade), bis(2-ethylhexyl)phosphoric acid (95%) and heptane fraction from petroleum (laboratory reagent grade) were purchased from Thermo Fisher Scientific (Merelbeke, Belgium). Cobalt(ii) sulfate heptahydrate (99+%) and molecular sieves (3 Å) were obtained from Acros Organics (Geel, Belgium). Copper(ii) sulfate pentahydrate (≥98%) was procured from Merck nv (Overijse, Belgium). Nickel(ii) sulfate hexahydrate (p.a.) was purchased from Th. Geyer GmbH (Renningen, Germany). Ionquest 290 was procured from Italmatch Chemicals (Genova, Italy). Mextral P507 and Mextral 54-100 were obtained from Kopper Chemical Industry Corp. (Chonqing, China). LIX 84-I was purchased from Syensqo SA (Beveren, Belgium). Bis(2,4,4-trimethylpentyl)dithiophosphinic acid was synthesized according as reported previously (“medium purity” protocol).^14^ Note that this compound is referred to by its commercial name (Cyanex 301) throughout, although the used sample was not the discontinued commercial extractant. All other chemicals were used as received. Water used for analysis was always of ultrapure quality, purified to a TOC of <2 ppb and deionized to a resistivity of 18.2 MΩ cm by a Millipore Milli-Q Reference® system.

### UV-VIS-NIR absorption spectroscopy

UV-VIS-NIR absorption spectra were recorded between 250 and 1350 nm on a Agilent Cary 6000i spectrophotometer at a resolution of 1 nm, and processed using the Cary WinUV software package. Quartz glass cuvettes with a path length of 1, 2 or 10 mm were used. The barren extractant solution was used as reference sample, to account for absorption by impurities present in the neat extractant.

Samples of the loaded extractant for spectroscopic studies were prepared by contacting a 5 mL aliquot of a 1 mol L^−1^ solution of the extractant in heptane with an equal volume of water, containing 0.2 equivalents of NaOH and 0.1 equivalents of the sulfate salt of the desired metal ion. Samples were shaken overnight in 20 mL glass screwcap vials. To prevent entrainment of aqueous droplets in the organic phase, the samples were centrifuged prior to spectroscopic analysis.

## Results and discussion

### Theory

The metal–extractant complex is a coordination compound between the metal ion and the extractant, which acts as an inner-sphere ligand with direct metal–extractant bonds. These bonds are largely electrostatic in nature, and hence the charge density of the metal ion chiefly determines the stability of the complex. As a result, higher-valent metal ions are generally preferentially extracted over lower-valent metal ions by acidic extractants. However, the metal–ligand bonds also have a non-negligible degree of covalent character, which can result in additional stabilization of the complex. Molecular orbital theory can be applied to coordination complexes (*ligand field theory*). Ligand field theory predicts that symmetry adapted linear combinations (SALCs) of electron-donating ligand orbitals decrease in energy upon complexation, acting as binding orbitals. Meanwhile, the d-orbitals of the metal ion either take on non-bonding or antibonding character, depending on the coordination polyhedron.^15–17^ These calculations are relatively complex, but a simple, semiquantitative alternative exists in the form of *crystal field theory*. This theory models the loss of degeneracy of the d-orbitals in a coordination complex as a simple electrostatic perturbation by point charges, which are centered at the ligand binding sites.^16–18^ Thus, the stability of the complex is now governed by two electrostatic effects: (1) an isotropic contribution, dependent on the charge density of the central metal cation and (2) a non-isotropic contribution, due to differential repulsion experienced by the d-orbitals in the presence of localized, electron-rich ligands. This electrostatic model of d-orbital splitting has successfully been applied to explain spectroscopic properties,^18–20^ hydration energies,^15,21,22^ magnetic properties^23,24^ and stability trends^25,26^ in transition-metal complexes.

**Fig. 2:**
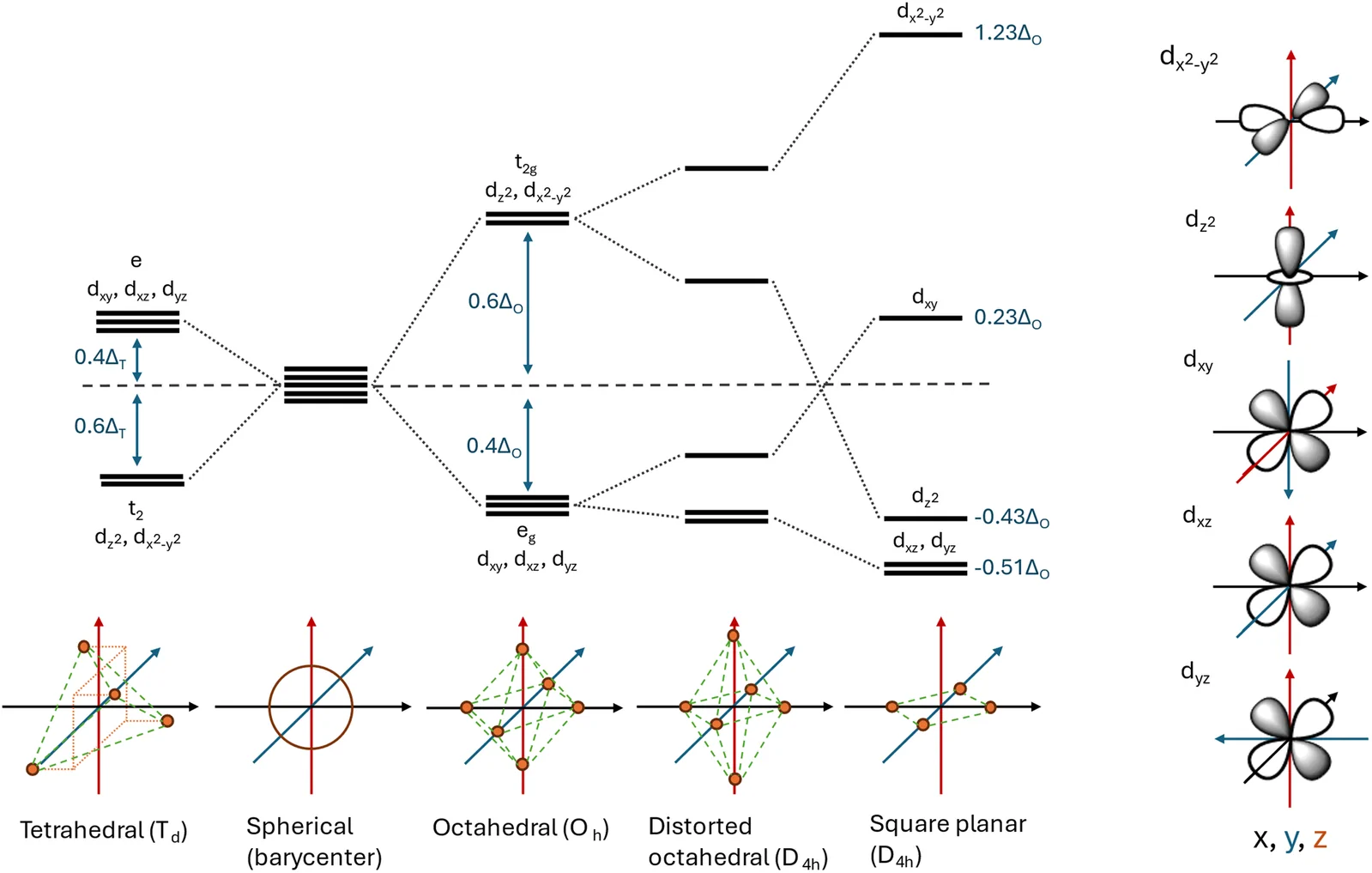
Splitting of the 5 d-orbitals in crystal fields of various symmetries.^27^ The relative separations for the *D*_4h_ symmetric geometries are approximate.

Crystal field theory adopts the reasoning that electrons in d-orbitals oriented towards the ligands experience more electrostatic repulsion than those oriented away.^17^ This electrostatic repulsion models antibonding covalent interactions. The five d-orbitals thus split differently in various crystal fields of different symmetry. Tetrahedral (*T*_d_), octahedral (*O*_h_), axially-distorted octahedral (*D*_4h_) and square-planar fields (*D*_4h_) are the most commonly encountered, in particular in the context of hydrometallurgy. As is evident from the corresponding point groups, the splitting is qualitatively similar in axially-distorted octahedral and square-planar fields, but the magnitude will be greater in square-planar fields due to the complete absence of axial ligands. Fig. 2 shows how the d-orbitals split in each one of these fields, with respect to a fully isotropic (spherical) electric field (barycenter).^27^ The relative separations given for the distorted octahedral and square-planar fields are approximate and depend on the ratio of the radius of the metal ion to the metal–ligand bond distance.

The magnitude of the splitting is quantified by the crystal field splitting parameter *Δ* (Fig. 2). Crystal field theory does not offer an explanation as to the value of this parameter. Instead, ligands are ranked in an empirical spectrochemical series, derived from their absorption spectra*.*^28–30^ Similarly, a spectrochemical series exists for the metal ions, with higher-valent metal ions having a larger value for *Δ*.^29,30^ Ligand field theory accounts for differences in splitting magnitude by considering the effects of π-bonding between ligands and the otherwise nonbonding d-orbitals.^15–17^ Crystal field theory does predict that, for the same combination of metal ion and ligand, the value of *Δ* for a tetrahedral complex is about −4/9 of that for an octahedral complex (*Δ*_T_ ≈ 4/9*Δ*_O_).^19^ This value assumes that bond distances are invariable between the two geometries, while in practice reduced steric strain may result in smaller bond distances in a tetrahedral complex, in particular with bulky extractants a ligands. Hence, this value should be treated as approximate.

The covalent stabilization of the complex can be estimated by assigning the central metal ion's d-electrons to the d-orbitals of known energy. In a degenerate set of d-orbitals, Hund's rule is always valid, and electrons are spread over orbitals in order to obtain the highest possible number of parallel spins in the ground state. Hund's rule may be voided if the degeneracy is lifted in a crystal field. This occurs if the energy difference between two levels (*Δ*) is greater than the energy required to pair two electrons (strong field regime).^27,31,32^ For octahedral complexes of divalent metals, however, this only occurs in combination with very strong-field ligands such as cyanide ions. This is not observed for common extractants as ligand (*vide infra*), and will thus not be considered. Tetrahedral complexes can be assumed to obey Hund's rule due to the small value of *Δ*_T_. Hund's rule will thus always be assumed to be in effect in this discussion.

**Fig. 3:**
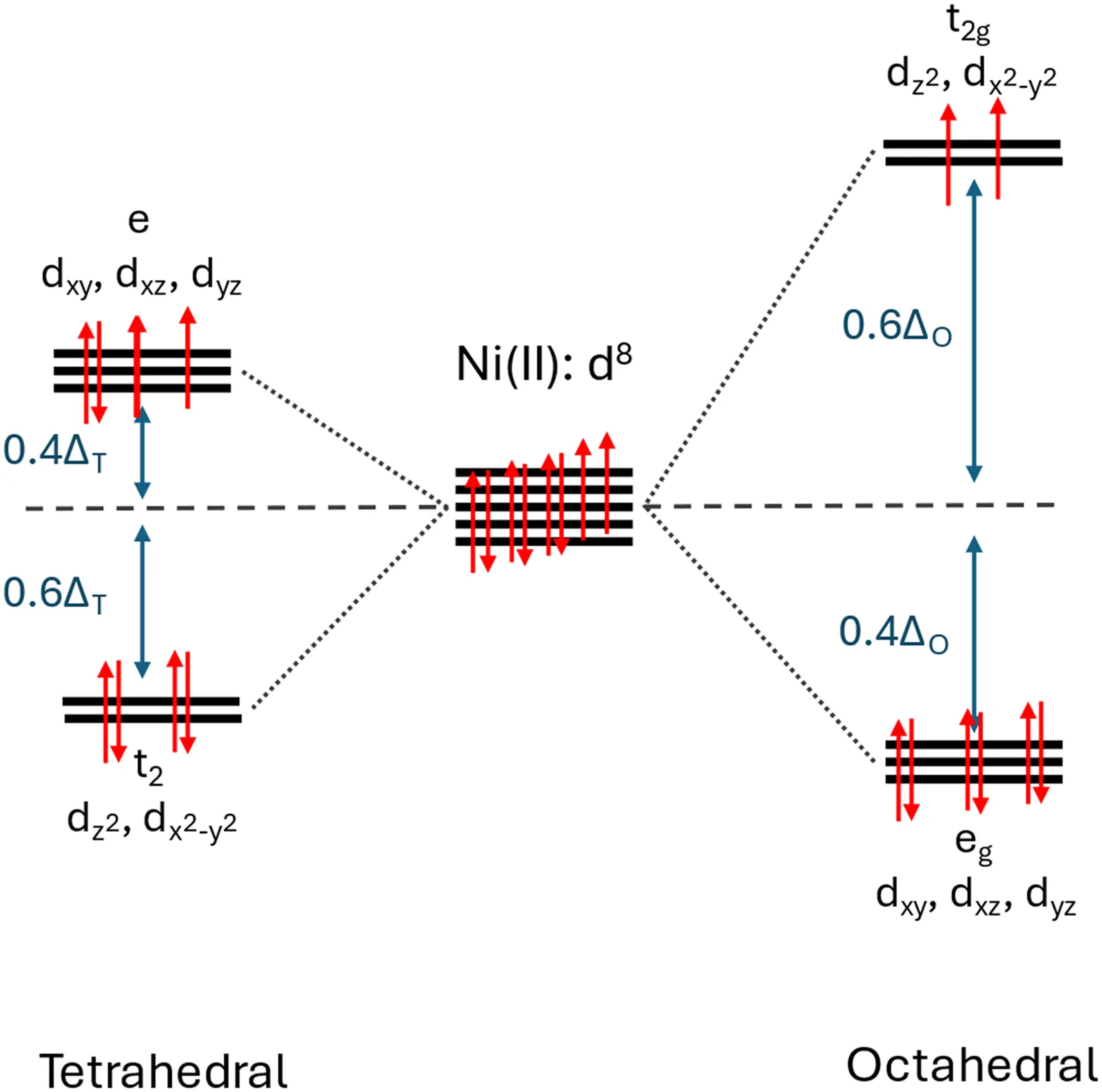
Electron configuration of Ni(ii) in a tetrahedral *vs.* octahedral crystal field.

The total number of d-electrons can be derived by subtracting the ion's charge from the group number in the periodic system (IUPAC numbering). Electrons can then be distributed over the split d-orbitals as described above. With this information, the covalent contribution to the total energy can be estimated as a function of *Δ*. This contribution is known as the ligand field stabilization energy (LFSE). For example, Ni(ii) (8 d-electrons, *i.e.* d^8^ ion), has a LFSE of −1.2*Δ*_O_ in an octahedral field, and −0.8*Δ*_T_ (−0.36*Δ*_O_) in a tetrahedral field (Fig. 3). This results from simple addition of the energies pertaining to each electron in the scheme (eqn (2) and (3)):

2 LFSE_Ni(ii),O_ = 6 × (−0.4*Δ*_O_) + 2 × (0.6*Δ*_O_) = −1.2*Δ*_O_

3 LFSE_Ni(ii),T_ = 4 × (−0.6*Δ*_T_) + 4 × (0.4*Δ*_T_) = −0.8*Δ*_T_ ≈ −0.36*Δ*_O_

**Fig. 4:**
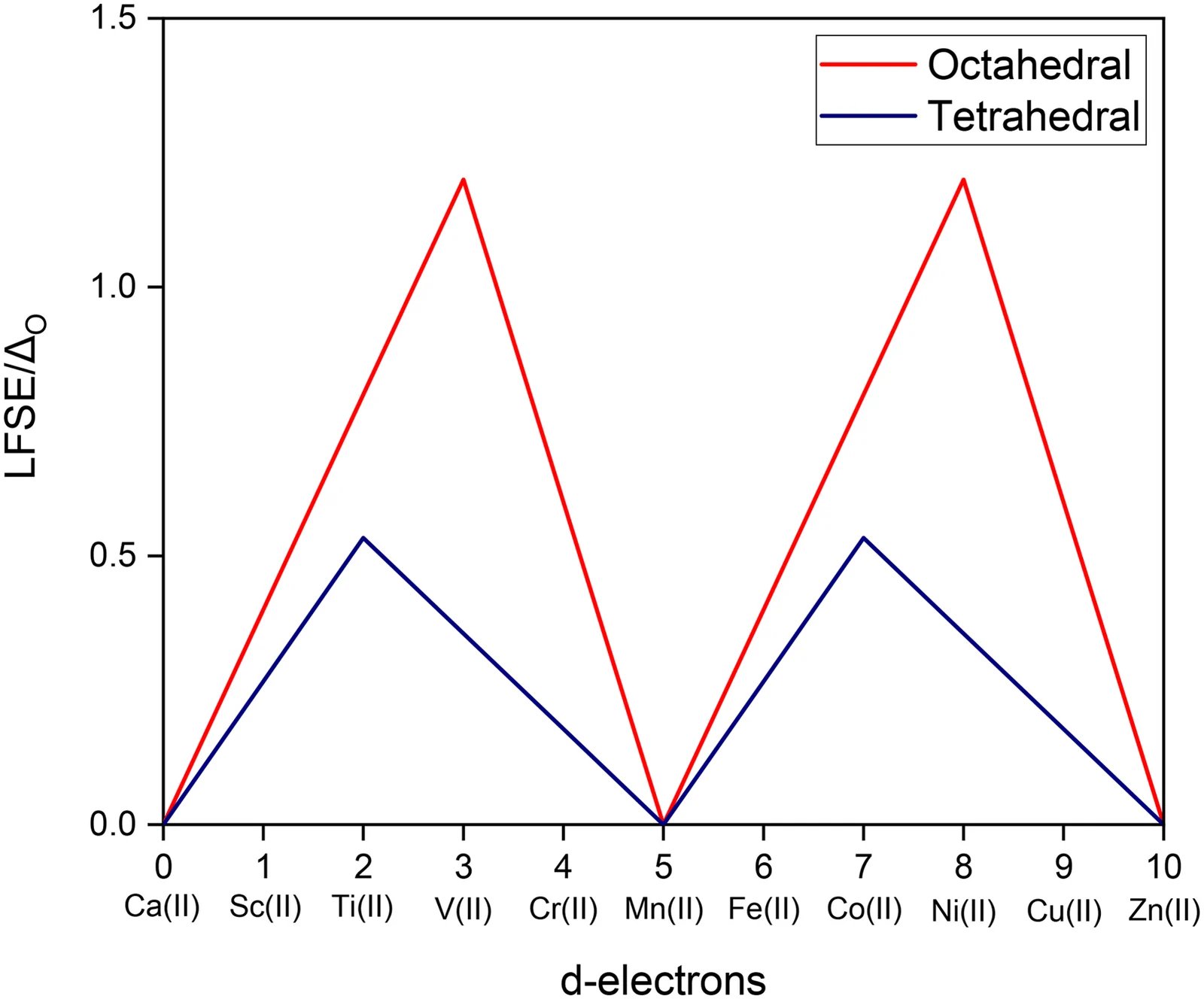
LFSE (expressed in terms of *Δ*_O_) for tetrahedral and octahedral complexes of divalent fourth-period transition metal ions.

This calculation can be carried out for all divalent metal ions of the fourth period. The result shows that octahedral d^3^ and d^8^ ions, *i.e.* V(ii) and Ni(ii) have the strongest LFSE contribution, as well as the largest difference in LFSE between the octahedral and tetrahedral geometry (Fig. 4). This results in their well-known tendency to adopt an octahedral configuration. Note that for Ca(ii), Mn(ii) and Zn(ii), which are respectively d^0^, d^5^ and d^10^ ions, electrons are evenly divided over all d-orbitals, resulting in a net zero LFSE.

The LFSE exists for both the aqueous ion, present as an octahedral complex with six water molecules as ligands, and for the organic phase ion, present as a complex with the extractant (and/or water) of arbitrary geometry.^33^ The net energy gain or loss from the LFSE contribution will thus be the difference between the LFSE of the organic phase complex and that of the hydrated ion.

This leads to three possible scenarios. The first scenario is that where the extractant is a stronger field ligand than water, *i.e.* it is located higher in the spectrochemical series. *Δ*_O_ will be greater in the organic phase than in the aqueous phase, and the LFSE will be greater after extraction. The net covalent effect upon extraction will be stabilizing. This effect is zero for Ca(ii), Mn(ii) and Zn(ii), but large for Ni(ii). Note that Ni(ii) can be expected to retain octahedral configuration in the organic phase due to the large LFSE advantage of this geometry for a d^8^ ion. This is supported by experimental evidence (*vide infra*). For Cu(ii) and Co(ii), the LFSE has an intermediate value. Hence, these extractants can be expected to exhibit a preference for metal ions with a large LFSE per *Δ*, extracting in the order Ni(ii) > Cu(ii), Co(ii) > Zn(ii), Mn(ii), Ca(ii).

The second scenario occurs for extractants that lie low on the spectrochemical series, and that thus have a smaller value for *Δ* than water. Hence, there is a net loss in LFSE upon extraction, due to a decrease of *Δ*. This loss is zero for Ca(ii), Mn(ii) and Zn(ii), but large for Ni(ii). As a result, these extractants will preferentially extract metals with zero LFSE. The observed order of extraction is thus reversed with respect to the first scenario: Zn(ii), Ca(ii) < Cu(ii), Co(ii) < Ni(ii).

In the third and final scenario, *Δ* is similar in magnitude for the species in both phases. Hence, the influence of the LFSE change will be negligible to that of the isotropic electrostatic contribution. The anionic, deprotonated extractant in the organic phase will preferentially bind to ions with a high charge density. As a consequence, the expected order of extraction is the Irving–Williams series: Ca(ii) ≪ Mn(ii) < Co(ii) < Ni(ii) < Cu(ii) > Zn(ii).^34^

The anomaly observed for Cu(ii) in the Irving–Williams series is a result of the so-called Jahn–Teller effect, a distortion of the octahedral coordination geometry when degenerate orbitals are asymmetrically occupied.^35^ This effect is particularly strong for octahedrally configured d^9^ ions, which results in exceptional stability for their complexes due to reduced equatorial bond distances.^17^ It should be noted that Cu(ii) may thus be extracted preferentially, even if this does not accord with the LFSE principles outlined above.

These three scenarios may be termed as the “strong field”, “weak field” and “intermediate field” regimes. It is important to mention, however, that this is a classification relative to the field strength of water as a ligand, where “intermediate field” pertains to an extractant with a field strength similar to water. In more general terms, the term “strong field ligand” is often used in the literature to refer to ligands which cause a change in spin multiplicity with respect to the gas-phase ion, and is thus a different definition from that which is employed in the context of this work.

While the geometry of Co(ii) can be variable in the organic phase, the influence of the geometry on the extraction sequence is expected to be limited. As is evident from Fig. 7, the LFSE of a Co(ii) ion is intermediate for both the octahedral and tetrahedral geometry. The tetrahedral geometry is favored for more sterically hindered ligands with a low value for *Δ*. For extractants with a low value of *Δ*, transition to a tetrahedral geometry will lead to a further loss of LFSE as compared to the hydrated ion. However, this may be fully compensated by a reduction in steric hindrance, as it is a reduction in steric hindrance and charge buildup that usually drives the formation of tetrahedral complexes. As will be discussed later, empirical observations of the spectrochemical series for octahedral complexes appear to allow prediction of the behavior of Co(ii) irrespective of its coordination geometry.

In the following section, spectroscopic studies will be used to determine *Δ* for various metal–extractant combinations. These values will be used to classify extractants according to the three scenarios enumerated above, and to rationalize their observed selectivity series.

### Spectroscopic studies

The scheme shown in Fig. 3 may at first glance appear to suggest that only one d–d transition exists in tetrahedral and octahedral transition metal complexes. This is true for d^1^ and d^9^ ions, but all other electronic configurations give rise to multiple transitions in the UV-VIS-NIR spectral region.^20,32^ Even in the absence of a crystal field, the gas phase ion exhibits several excited states which differ in energy due to the different spatial distribution of the d-orbitals (and hence differences in electron repulsion between various configurations) and spin–spin coupling (with lower spin multiplicities being higher in energy). In a first-order approximation, the energy differences between these states are quantified by the Racah parameters *B* and *C*.^20^ The gas-phase terms split in the presence of the crystal field, leading to two types of transitions: (1) pure crystal-field transitions, which arise from a split of the gas-phase ground state term, and (2) transitions to levels splitting from excited state terms of the gas phase ion. The former have an energy corresponding only to a multiple of *Δ*, while the latter depend on *Δ* as well as the Racah parameters.^20^ As the Racah parameters can change in the presence of ligands due to the nephelauxetic effect (electron cloud expansion resulting in reduced repulsion), determining *Δ* from these is not trivial.^30,36^ It is thus paramount to identify the features in the spectrum which correspond to pure crystal field transitions (with energy *nΔ*, where *n* corresponds to the number of electrons involved in the transition). This identification can be done using Tanabe–Sugano diagrams as a guide.^32^

Assuming identical coordination modes and geometries between the various metal ions for each extractant, the spectrochemical series of the extractants is would be identical for each metal ion. This would allow the use of a single metal ion as probe to determine the series, which could be applied to all other metal ions. In practice, the speciation is not entirely identical between various metal ions, leading to changes in the spectrochemical series for various metal ions. Hence, three metal ions were used as probes: Cu(ii), Ni(ii) and Co(ii), each of which have suitable transitions in the UV-VIS-NIR range for the determination of *Δ*. Evidently, the absence of d-d transitions in Ca(ii) and Zn(ii) precludes their inclusion in the study, while the d-d transitions of Mn(ii) are subject to strong configuration interaction, making them nearly independent of *Δ* in the weak-field limit.^32^ Each of these ions has a LFSE of 0, however, and knowledge of *Δ* is thus moot in the prediction of the stability of their complexes. As will be discussed below, while small differences in the spectrochemical series are observed between metal ions, the general division into strong, intermediate and weak-field ligands remains applicable.

**Fig. 5:**
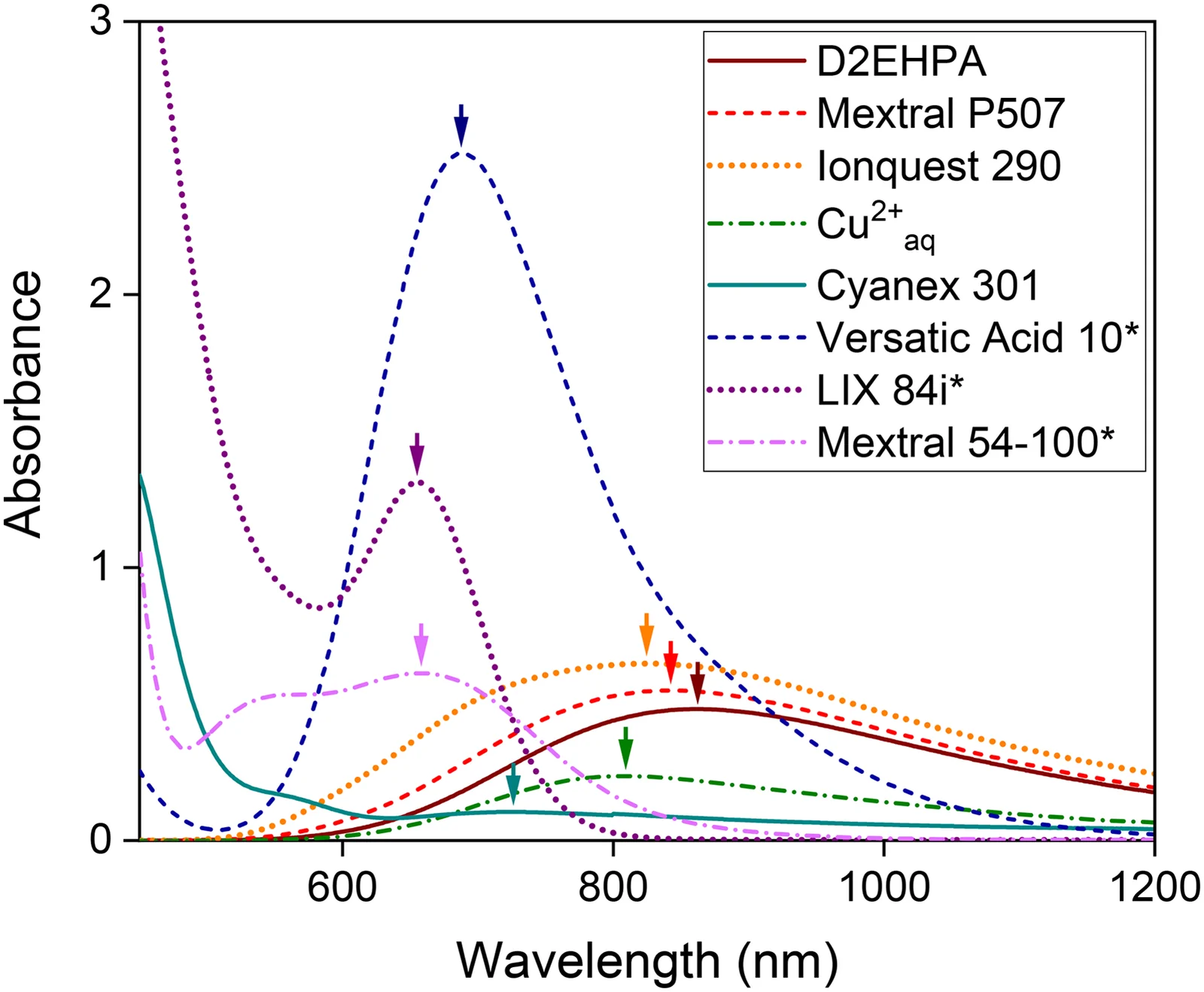
UV-VIS-NIR absorption spectra of 1 mol L^−1^ solutions of extractant in heptane, at 20% loading with Cu(ii). Extractants labeled with an asterisk were measured in a cell with 1 mm path length, other extractants were measured in a 2 mm cell.

In d^1^ and d^9^ ions, all states have the same net electron repulsion and spin multiplicity. For the d^9^ Cu(ii) ion, it is thus trivial to identify the band corresponding to the pure crystal-field transition, with only the ^2^E_g_ → ^2^T_2g_ band present in the spectrum of octahedral species. Hence, Cu(ii) was chosen as the first probe element for spectroscopic studies. The UV-VIS-NIR absorption spectra of 1 mol L^−1^ solutions of extractant, at 20% loading with Cu(ii) are shown in Fig. 5.

All complexes, except those formed by Cyanex 301 and Mextral 54-100, show the typical single absorption band of an octahedral d^9^ complex. Tetrahedral complexes also exhibit a single band, but at significantly lower energy (*Δ*_T_ ≈ 4/9*Δ*_O_). The intense bands often observed in the UV region at short wavelength (*λ*) are attributable to ligand-to-metal charge transfer bands. These features are characterized by their high intensity as charge transfer transitions are not subject to the Laporte selection rule, which forbids d-d transitions in the absence of perturbations.^37^

The Cyanex 301 complex shows a very strongly attenuated d-d transition (*λ*_max_ = 725 nm). This was also observed by Sole and Hiskey, who additionally reported a loss of paramagnetism.^38^ The authors attributed these observations to the reduction of Cu(ii) to Cu(i) by the extractant, which in turn is oxidized to the disulfide derivative. The d^10^ Cu(i) ion exhibits no d-d transitions, and the absorption below 600 nm is likely due to a metal-to-ligand charge transfer band (MLCT).

Mextral 54-100 is the only extractant with a distinct splitting of the d-d band of its Cu(ii) complex, indicating a symmetry lowering of the crystal field to *D*_4h_. The small separation between these bands points to an octahedral geometry with significant axial elongation. The transition at the lowest energy (672 nm) corresponds, irrespective of the degree of deviation from the octahedral structure, to the d_*xy*_ → d_*x*^2^–*y*^2^_ transition with a transition energy equal to the value of *Δ*_O_ for an idealized octahedral field. In the square planar crystal field limit, a band at 395 nm would be predicted for the d_*x*^2^_ → d_*x*^2^–*y*^2^_ transition. A bimodal Voigt deconvolution was applied in order to extract the value of *Δ*_O_ from the maximum of the lowest energy band (see Fig. S1), while the second band allowed predicting the energy of the other d-orbitals.

**Fig. 6:**
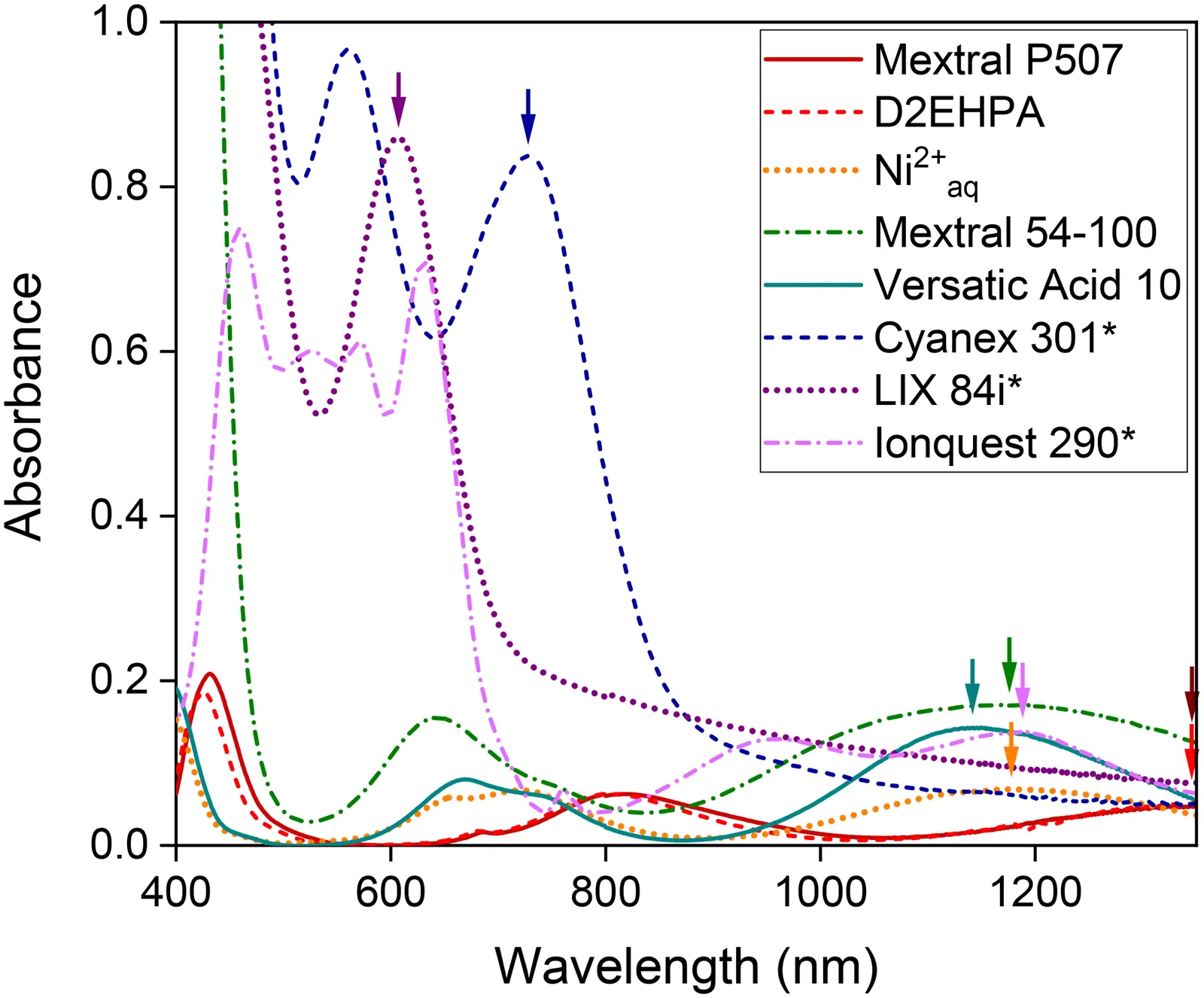
UV-VIS-NIR absorption spectra of 1 mol L^−1^ solutions of extractant in heptane, at 20% loading with Ni(ii). Extractants labeled with an asterisk were measured in a cell with 1 mm path length, other extractants were measured in a 2 mm cell.

Fig. 6 shows the spectra of extractant samples (1 mol L^−1^ in heptane) at 20% loading with Ni(ii). Note that the samples for D2EHPA, Mextral 507 and Ionquest 290 were dried over molecular sieves prior to measuring, as it is known from earlier studies that the extractant may, partly of fully, reside in the second coordination sphere, with water molecules occupying the inner sphere.^33^ Ni(ii) is a d^8^ ion, and as such the spectrum is convoluted due to the existence of states splitting from non-degenerate zero-field terms. The Tanabe-Sugano diagram for octahedral d^8^ complexes must thus be used to identify the band with an energy corresponding to *Δ*.^32^ All spectra, except that of loaded Ionquest 290, are consistent with octahedral symmetry. The lowest energy band corresponds to the ^3^A_2g_ → ^3^T_2g_(F) transition, with both states splitting from the zero-field ground state term. The transition thus has an energy of *Δ* (this value is approximate due to assumptions made in the Tanabe–Sugano model). For the tetrahedral complex with Ionquest 290, the Tanabe–Sugano diagram for octahedral d^2^ complexes can be used to identify the bands, as the description of a d^2^ ion in an octahedral field is mathematically equivalent to that of a d^8^ ion in a tetrahedral field.^39^ The weak, spin-forbidden band (*λ*_max_ = 760 nm) corresponding to a triplet-to-singlet intersystem crossing can be used to unambiguously identify the geometry as tetrahedral, implying that the band immediately to longer wavelengths, must correspond to the ^2^T_1_ → ^3^A_2_ transition with an energy of approximately 2*Δ*_T_.^40^ The ^3^T_1_(F) → ^3^A_2_ band appears split (by 1.91⋯10^3^ cm^−1^), a feature which cannot be explained by spin-orbit coupling or symmetry reduction, as the ^3^A_2_ state is not orbitally degenerate.^41,42^ This behavior was also reported in a previous publication, where the band at higher energy was attenuated upon drying.^33^ This split is thus likely caused by the simultaneous occurrence of two species in equilibrium, under the influence of trace amounts of water. The lower-energy band thus corresponds to the structure pertinent in anhydrous conditions, *i.e.* the unperturbed Ni(ii)-Ionquest 290 complex in heptane. A deconvolution was applied to extract the exact position of the individual maxima of these overlapping bands (see Fig. S2).

In D2EHPA and Ionquest 290 systems, Ni(ii) is known to occur as a fully or partly hydrated ion in the organic phase at high loading, with one or both of the associated extractant anions in the second sphere.^33^ The coordination units have an octahedral geometry. The optical absorption spectra of solutions of this type were recorded earlier and are shown in the SI (Fig. S6). The ^3^A_2g_ → ^3^T_2g_(F) band of these species is located at a significantly shorter wavelength than for the corresponding anhydrous complexes, demonstrating the increase in *Δ* upon substitution of water for the extractant. Still, the empirical value of *Δ* remains smaller than that of hydrated Ni(ii).

**Fig. 7:**
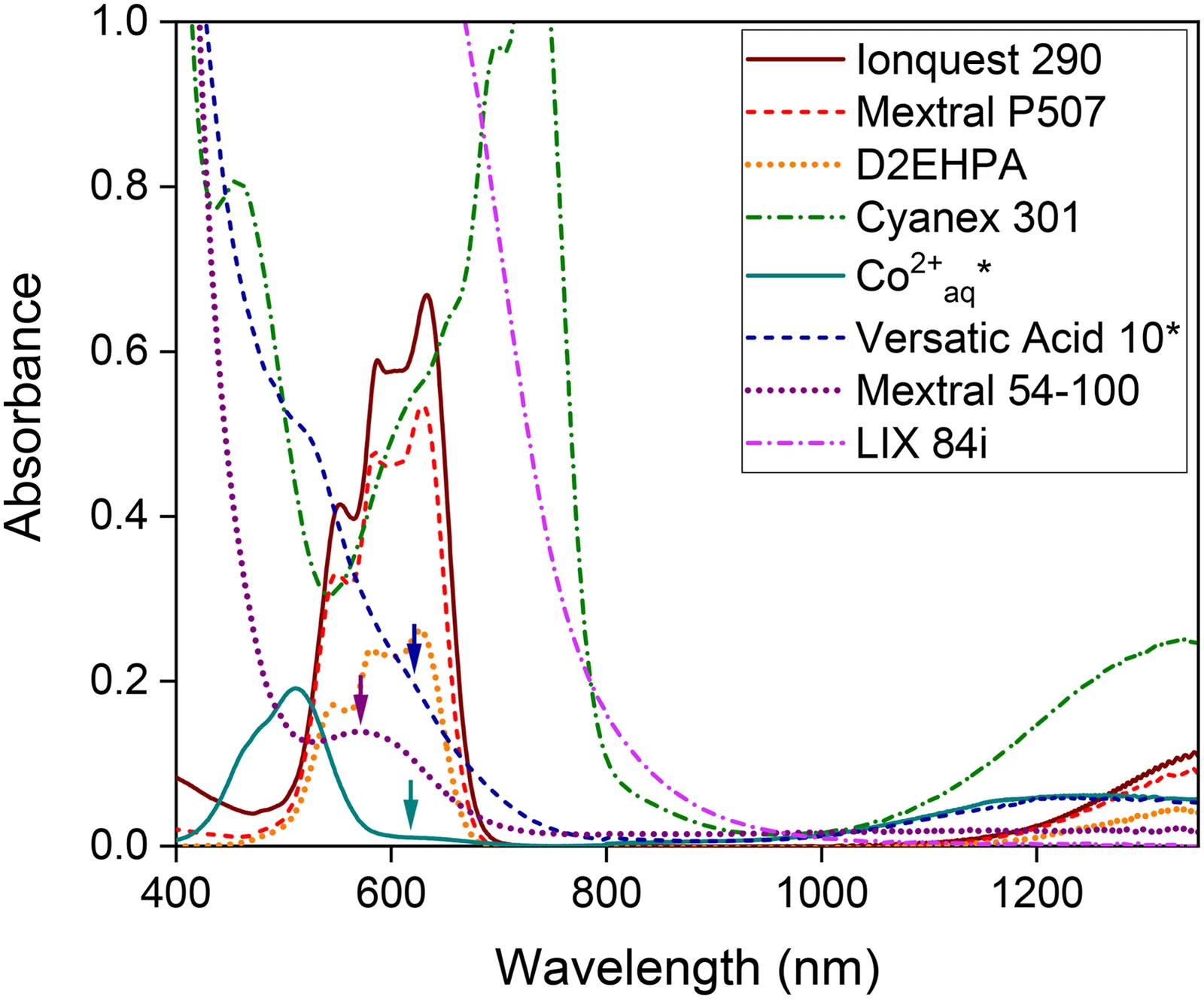
UV-VIS-NIR absorption spectra of 0.2 mol L^−1^ solutions of extractant in heptane, at 20% loading with Co(ii). Extractants labeled with an asterisk were measured in a cell with 10 mm path length, other extractants were measured in a 1 mm cell.

Finally, Fig. 7 shows the spectra of extractant samples (1 mol L^−1^ in heptane) at 20% loading with Co(ii). These spectra exhibit pervasive structural diversity, with tetrahedral coordination (dotted lines) for Ionquest 290, Mextral P507, D2EHPA and Cyanex 301, and octahedral coordination for Mextral 54-100, Versatic Acid 10 and aqueous Co(ii) sulfate. The spectrum of LIX 84-I is dominated by a very broad and intense charge transfer band, lacking resolved d-d transitions. Nonetheless, the absence of any d-d transitions below 800 nm excludes both a tetrahedral or a low-*Δ* octahedral complex, implying that LIX 84-I behaves as a relatively strong-field ligand. The tetrahedral structures show both a ^4^A_2_ → ^4^T_1_(F) transition with a maximum outside the range of measurement, and a ^4^A_2_ → ^4^T_1_(P) transition in the visible range (split by spin-orbit interactions).^41^ Unfortunately, the latter is not suitable for determining *Δ*, as the transition energy is also dependent on the (unknown) Racah parameters.^32^ In addition, as the ^4^T_1_(F) and ^4^T_1_(P) states are subject to mixing under the influence of the crystal field.^43^ Conversely, the value of *Δ* can easily be determined for the octahedral complexes, as the lowest-energy spin-allowed transition of these complexes is the ^4^T_1g_(F) → ^4^T_2g_(F) transition, with an energy corresponding to *Δ*. This transition is visible in all three octahedral species, but the location of the maximum is obfuscated by noise (caused by vibrational overtones of water) for aqueous Co(ii) and Co(ii) in Versatic Acid 10. For the Mextral 54-100 complex, this transition occurs at high energy (*λ*_max_ = 568 nm), allowing a convenient determination of *Δ*. For the Versatic Acid 10 complex and aqueous Co(ii), the ^4^T_1g_(F) → ^4^A_2g_(F) transition with an energy of 2*Δ* can be used, which appears as a shoulder on the low energy side of the ^4^T_1g_(F) → ^4^T_1g_(P) manifold. These overlapping bands must thus be deconvolved in order to extract a good estimate for *Δ*. This procedure is detailed in the SI (Fig. S3–S5).

Values for *Δ* were extracted from the spectral data as described above, and the resulting LFSE values were calculated and tabulated in Table 1. In addition, the difference in LFSE is given between the ion in the extracted complex and the hydrated ion in the aqueous phase. All energies are expressed as wavenumbers (ν). The relative positions of the extractants on the spectrochemical series are largely conserved across the evaluated metal ions, save for a few exceptions. For example, Mextral 54-100 is a relatively strong field ligand for Cu(ii) and Co(ii), but remarkably weaker for Ni(ii). In addition, the relative positions of Cyanex 301 and Versatic Acid 10 are reversed for Ni(ii) as compared to Cu(ii). These differences are attributable to differences in the coordination mode of the extractant, including distortion of the coordination geometry, monomeric *vs.* dimeric coordination, monodentate *vs.* bidentate coordination, the formation of mononuclear *vs.* bridged polynuclear complexes, and the saturation of the coordination sphere with either water molecules or additional (neutral) extractant molecules.^33^

**Table 1:** Values for *Δ* and ligand field stabilization energy for the evaluated metal ion–extractant pairs, as well as the difference in LFSE upon extraction from an aqueous sulfate solution

Metal	Extractant	Geometry^a^	Transition	Value	*λ*_max_ (nm)	*ν*_max_ (10^3^ cm^−1^)	*Δ* (10^3^ cm^−1^)	LFSE (10^3^ cm^−1^)	*Δ*LFSE_water_^b^ (10^3^ cm^−1^)
Cu(ii)	LIX 84-I	*O*_h_	^2^E_g_ → ^2^T_2g_	*Δ*_O_	656	15.2	15.2	9.15	1.73
Cu(ii)	Mextral 54-100	*D*_4h_	d_*z*^2^_ → d_*x*^2^–*y*^2^_	*Δ*_O_	672^g^	14.9	14.9	9.64^c^	2.23
Cu(ii)	Versatic acid 10	*O*_h_	^2^E_g_ → ^2^T_2g_	*Δ*_O_	677	14.5	14.5	8.72	1.30
Cu(ii)	Cyanex 301^d^	*O*_h_	^2^E_g_ → ^2^T_2g_	*Δ*_O_	723	13.8	13.8	8.30	0.88
Cu(ii)	Water	*O*_h_	^2^E_g_ → ^2^T_2g_	*Δ*_O_	809	12.4	12.4	7.42	0
Cu(ii)	Ionquest 290	*O*_h_	^2^E_g_ → ^2^T_2g_	*Δ*_O_	825	12.1	12.1	7.27	−0.14
Cu(ii)	Mextral P507	*O*_h_	^2^E_g_ → ^2^T_2g_	*Δ*_O_	844	11.8	11.8	7.11	−0.31
Cu(ii)	D2EHPA	*O*_h_	^2^E_g_ → ^2^T_2g_	*Δ*_O_	861	11.6	11.6	6.97	−0.45
Ni(ii)	LIX 84-I	*O*_h_	^3^A_2g_ → ^3^T_2g_	*Δ*_O_	607	16.5	16.5	19.8	9.6
Ni(ii)	Mextral 54-100	*O*_h_	^3^A_2g_ → ^3^T_2g_	*Δ*_O_	1167	8.57	8.57	10.3	0.1
Ni(ii)	Versatic acid 10	*O*_h_	^3^A_2g_ → ^3^T_2g_	*Δ*_O_	1143	8.75	8.75	10.5	0.3
Ni(ii)	Cyanex 301	*O*_h_	^3^A_2g_ → ^3^T_2g_	*Δ*_O_	727	13.8	13.8	16.5	6.3
Ni(ii)	Water	*O*_h_	^3^A_2g_ → ^3^T_2g_	*Δ*_O_	1177	8.50	8.50	10.2	0
Ni(ii)	Ionquest 290 (dry)^e^	*T*_d_	^3^T_1_ → ^3^A_2_	2*Δ*_T_	1178^g^	8.49	4.24	3.40	−6.80
Ni(ii)	Mextral P507 (dry)^e^	*O*_h_	^3^A_2g_ → ^3^T_2g_	*Δ*_O_	≈1135^f^	≈7.4	≈7.4	≈8.9	≈−1.3
Ni(ii)	D2EHPA (dry)^e^	*O*_h_	^3^A_2g_ → ^3^T_2g_	*Δ*_O_	≈1135^f^	≈7.4	≈7.4	≈8.9	≈−1.3
Ni(ii)	Ionquest 290 (wet)	*O*_h_	^3^A_2g_ → ^3^T_2g_	*Δ*_O_	1258	7.95	7.95	9.54	−0.66
Ni(ii)	D2EHPA (wet)	*O*_h_	^3^A_2g_ → ^3^T_2g_	*Δ*_O_	1228	8.14	8.14	9.77	−0.42
Co(ii)	Mextral 54-100	*O*_h_	^4^T_1**g**_ → ^4^T_2**g**_	*Δ*_O_	572	17.5	17.5	13.4	7.4
Co(ii)	Versatic acid 10	*O*_h_	^4^T_1**g**_ → ^4^A_2**g**_	2*Δ*_O_	577^g^	17.3	8.67	6.93	0.39
Co(ii)	Water	*O*_h_	^4^T_1**g**_ → ^4^A_2**g**_	2*Δ*_O_	611^g^	16.4	8.18	6.55	0

a Point group of the crystal field only.

b Net ligand field stabilization upon extraction from an aqueous sulfate solution.

c Calculation elaborated in SI (page S10).

d Residual Cu(ii) signal used.

e Dried overnight over 3 Å molecular sieves.

f Peak near edge of measurement range.

g Peak maximum obtained from spectral deconvolution with uncertainty of approx. 2% on both *λ*_max_ and derived quantities.

Determining the LFSE for undistorted octahedral and tetrahedral complexes is straightforward (Fig. 4). For the axially elongated octahedral complex of Cu(ii) with Mextral 54-100, an additional calculation is necessary. The degree of axial distortion can be determined from the relative energies of the d_*z*^2^_ to d_*x*^2^–*y*^2^_ and d_*zy*_ to d_*x*^2^–*y*^2^_ transitions. This ratio varies between 1 for an unperturbed octahedron, and 1.66 for four-coordinate square planar geometry (Fig. 2). The full calculation is shown in the SI (page S10).

The data in Table 1 can now be used to classify extractants according to the scheme outlined above. LIX 84-I has the largest values for *Δ* of all evaluated extractants and thus occupies the highest position on the spectrochemical series. Its reported order of extraction is Cu(ii) > Ni(ii) > Co(ii) > Zn(ii) > Mn(ii), ordered from low to high equilibrium pH.^5,44^ The Jahn–Teller effect accounts for the efficient extraction of Cu(ii), while the other metals are ordered according to the their LFSE/*Δ*_O_ ratio. Note that the value for *Δ*_O_ could not be determined exactly for Co(ii) due to interference from a LMCT band, but the absence of any electronic transitions below 800 nm indicates a relatively strong, octahedral crystal field, placing the LFSE for Co(ii) (0.80*Δ*_O_) between those of Ni(ii) (1.2*Δ*_O_) and Zn(ii)/Mn(ii) (LFSE = 0). The ordering of Zn(ii) and Mn(ii) can be attributed to their relative charge densities.

D2EHPA, Mextral P507 and Ionquest 290 all have values of *Δ* smaller than that of water. This results in an unfavorable change in LFSE upon extraction. Indeed, calculated values for the LFSE difference upon extraction are all negative. This explains the reported extraction sequences of the divalent metal ions of the fourth period for these extractants:^6^D2EHPA: Zn(ii) > Ca(ii) > Mn(ii) > Cu(ii) > Co(ii) > Ni(ii)Mextral P507/PC-88a/Ionquest 801: Zn(ii) > Ca(ii) ≈ Cu(ii) ≈ Mn(ii) > Co(ii) > Ni(ii)Ionquest 290/Cyanex 272: Zn(ii) > Cu(ii) > Mn(ii) > Co(ii) > Ca(ii) > Ni(ii)D2EHPA selectively extracts ions with zero LFSE at low pH: Zn(ii), Ca(ii) and Mn(ii). Zn(ii) is likely extracted the most strongly of this group because of its high charge density, while Ca(ii) is known to accommodate a higher number of extractant molecules in its first coordination sphere than Mn(ii), providing additional stabilization of the ion in the organic phase.^33^ The elements with intermediate LFSE (and thus an intermediate loss of LFSE upon extraction) are extracted at moderate pH values. Cu(ii) is extracted before Co(ii), which could be a consequence of the Jahn–Teller effect. Finally, Ni(ii) is extracted only at high pH, as a very strong loss of LFSE is incurred when it is transferred from the aqueous phase into a complex with the extractant. In fact, the organic phase speciation is strongly shifted to the hydrated ion, with the extractant only present in the second sphere, as substitution of D2EHPA by water is highly favorable.

The sequences exhibited by Mextral P507 and Ionquest 290 are similar to that of D2EHPA and differ mostly in the positions of Cu(ii) and Ca(ii). Cu(ii) shifts towards more favorable extraction from the phosphoric to the phosphinic acid, which accords well with the rising value of *Δ* in this sequence of extractants. As the value of *Δ* increases, the unfavorable LFSE contribution upon extraction decreases, and the favorable Jahn–Teller effect begins to dominate. Hence, these extractants can be seen as intermediate between the weak-field and intermediate-field categories. Ca(ii) is extracted very poorly by Ionquest 290 compared to D2EHPA, which may be a result of the absence of the additional inner-sphere extractant molecule in the Ca(ii) complex of Ionquest 290.^33^

Cyanex 301 and Versatic Acid 10 are ranked intermediately on the spectrochemical series of the extractants. Their *Δ* values are sufficiently close to those of water that electrostatic effects largely negate the LFSE contribution. As a result, the observed extraction sequences follow the Irving–Williams series: Cu(ii) > Zn(ii) > Ni(ii) > Co(ii) > Mn(ii) > Ca(ii) for both extractants.^6^ As mentioned above, this series follows the charge density of the metal ions, with the exception of Cu(ii), which is subject to the Jahn–Teller effect.^34,35^ Note that this reasoning does not apply to Cu(ii) extraction by Cyanex 301, which largely occurs concomitantly with the reduction of copper to Cu(i) and the formation of a disulfide.^38^

Finally, Mextral 54-100 presents an intermediate case between the strong-field and intermediate-field extractant categories. This is because of a marked change in the *Δ* parameter of the extractant when bound to Ni(ii) as compared to Cu(ii) and Co(ii). This results in a reversal of the positions of Ni(ii) and Co(ii) with respect to the expected sequence for strong-field extractants, elucidating the unique extractant sequence observed for this extractant: Cu(ii) > Co(ii) > Ni(ii) > Zn(ii) > Mn(ii).^5^

The behavior of extractants appears to correlate well with the value of *Δ* found for the Cu(ii) complex. Cu(ii) is a convenient probe, as only one d-d transition exists, occurring at an energy of *Δ*. Mextral 54-100 is the only exception, as an uncharacteristically low value for *Δ* in the Ni(ii) complex results in unexpectedly weak extraction of this ion. It does not appear to be necessary to take the coordination geometry of Co(ii) into account. Although the transition to tetrahedral coordination for Cyanex 301, D2EHPA, Mextral P507 and Ionquest 290 leads to a large loss in LFSE upon extraction, Co(ii) is not extracted any more weakly than would be expected for an octahedral complex on the basis of the spectrochemical series. This can be rationalized by noting that the difference in LFSE for octahedral and tetrahedral coordination is relatively small for Co(ii) (Fig. 4). Moreover, tetrahedral coordination is usually driven by a reduction in steric hindrance, which outweighs the LFSE penalty in the overall energetic balance of a stable tetrahedral complex. The reduced favorable LFSE contribution is thus nullified by this decrease in unfavorable steric repulsion.

## Conclusions

A strong correlation was found between the position of an extractant on the spectrochemical series of ligands on the one hand, and the order in which the divalent metal ions of the fourth period of the periodic system are extracted on the other. Extractants which act as strong-field ligands tend to preferentially extract metal ions with a large ligand field stabilization energy (LFSE). Extractants positioned below water in the spectrochemical series preferentially extract ions with zero LFSE. Those which are positioned at intermediate positions, slightly above water, extract metal ions according to their charge density. These propensities can be rationalized in the framework of crystal field theory, by considering changes in ligand field stabilization energy upon extraction. Cu(ii) is often extracted the most strongly of all metal ions, because of the strong Jahn–Teller effect promoting complexation of the Cu(ii) ion. In general, Cu(ii) is a convenient probe to determine the relative position of an extractant on the spectrochemical series, although the relative position of extractants may vary slightly between metal ions.

## Conflicts of interest

There are no conflicts to declare.

## Supplementary Material

RE-011-D6RE00168H-s001

## Data Availability

The data supporting this article have been included as part of the supplementary information (SI). Supplementary information: spectral deconvolution, spectra of hydrated Ni(ii) samples, spectra of aged Co(ii) samples, calculation of LFSE in Cu(ii)–Mextral 54-100 sample, photographs of samples. See DOI: https://doi.org/10.1039/d6re00168h.
