# Supplementary material for: Pivotal influence of ligand field stabilization energy on the extraction order of divalent metal ions by acidic extractants
Source: React Chem Eng. 2026 Jun 15. Online ahead of print. doi: 10.1039/d6re00168h (PMC13289708; doi:10.1039/d6re00168h)
Supplement: RE-OLF-D6RE00168H-s001 [file RE-OLF-D6RE00168H-s001.pdf]

Supplementary Information (SI) for Reaction Chemistry & Engineering.
This journal is © The Royal Society of Chemistry 2026

Pivotal influence of ligand field stabilization energy on the extraction order of divalent metal ions by acidic extractants

Stijn Raiguel[†*] and Koen Binnemans[†]

[†] KU Leuven, Department of Chemistry, Celestijnenlaan 200F, P.O. box 2404, B-3001 Leuven, Belgium.

*Corresponding author:
Email: Stijn.raiguel@kuleuven.be

Supplementary Information

Table of contents

1. Spectral deconvolution S3
2. Spectra of hydrated Ni(II)-loaded extractants S8
3. Spectra of aged Co(II)-loaded samples S9
4. LFSE calculation for Cu(II)-Mextral 54-100 S10
5. Photographs of samples S11

1. Spectral deconvolution

Cu(II)–Mextral 54-100

A bimodal Voigt least square fit was applied to the absorption spectrum of Cu(II)-loaded Mextral 54-100 between 510 and 1000 nm. The cumulative fit had an adjusted R^2 value of 0.99999, with maxima at 533 and 672 nm for the individual components.

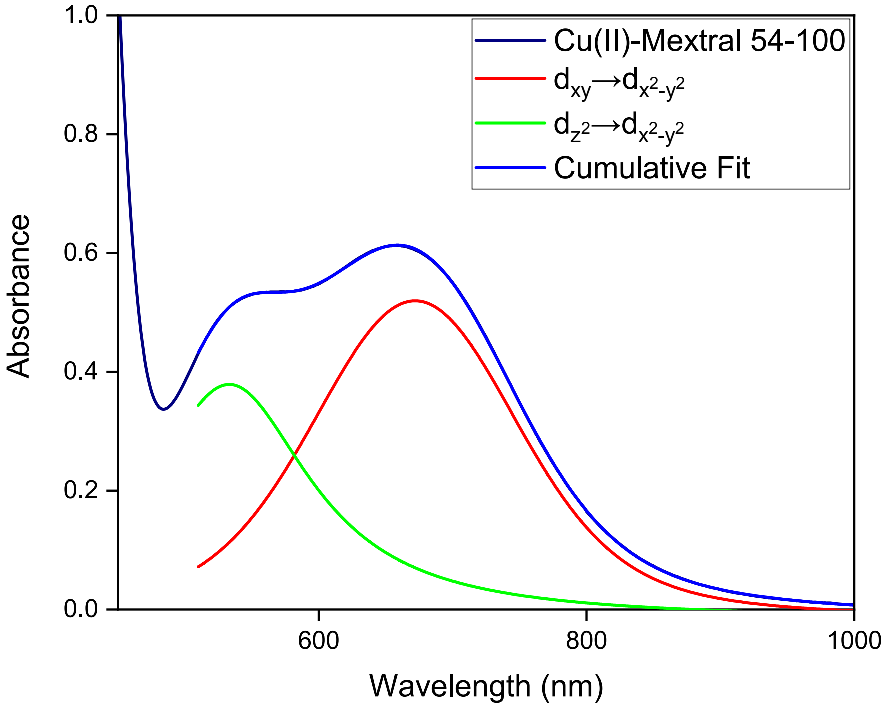

Fig. S1: Least square fit of two Voigt profiles to the absorption spectrum of Cu(II)-loaded Mextral 54-100 in the 510-1000 nm range.

Ni(II)–Ionquest 290 (dry)

A bimodal Voigt least square fit was applied to the absorption spectrum of Ni(II)-loaded Ionquest 290 (dried overnight over 3Å molecular sieves) between 800 and 1350 nm. The cumulative fit had an adjusted R^2 value of 0.99898, with maxima at 947 and 1178 nm for the individual components.

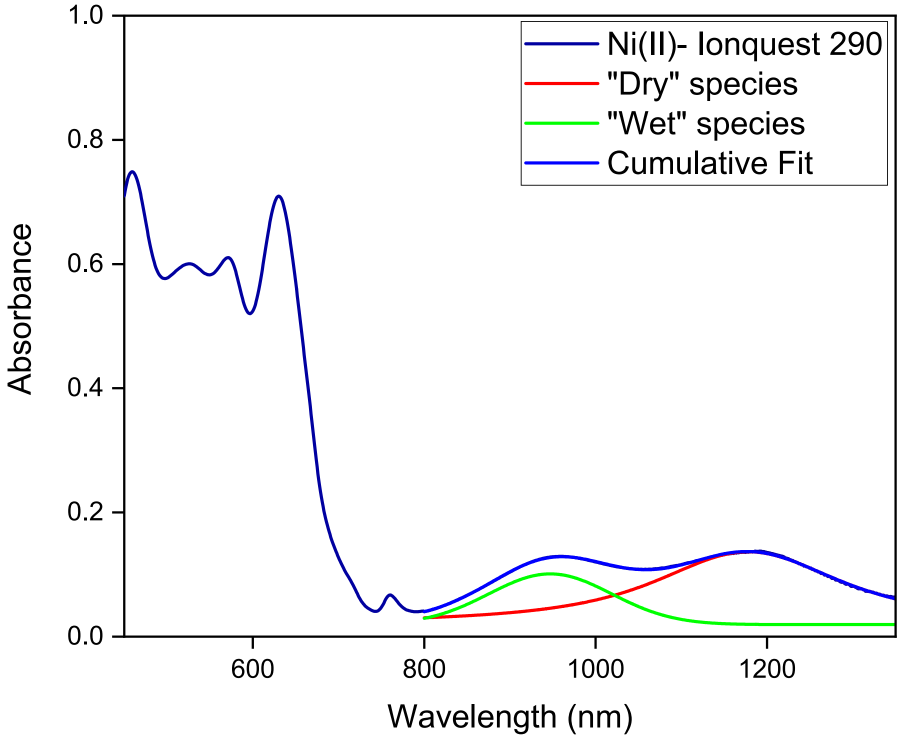

Fig. S2: Least square fit of two Voigt profiles to the absorption spectrum of Ni(II)-loaded Ionquest 290 (dried overnight over 3Å molecular sieves) in the 800-1350 nm range.

Aqueous Co(II) sulfate

A trimodal Voigt least square fit was applied to the absorption spectrum of aqueous Co(II) sulfate between 350 and 775 nm. The cumulative fit had an adjusted R^2 value of 0.99958, with maxima at 464 and 515 nm for the components of the $^4T_{1g}(F) \rightarrow {}^4T_{1g}(P)$ manifold and at 611 nm for the $^4T_{1g}(F) \rightarrow {}^4A_{1g}(F)$ transition.

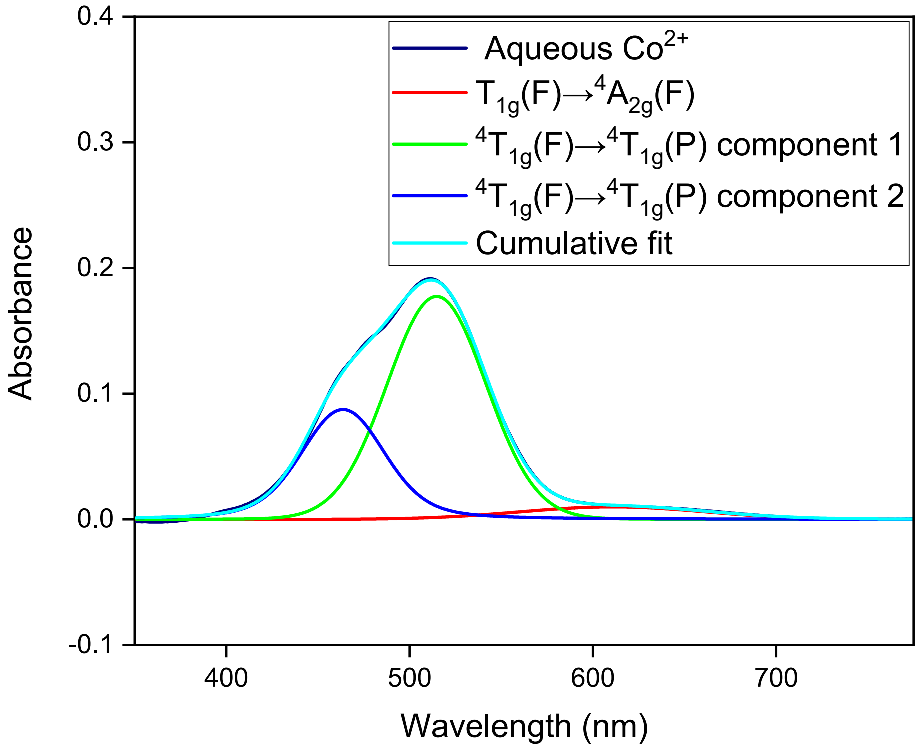

Fig. S3: Least square fit of three Voigt profiles to the absorption spectrum of aqueous Ni(II) sulfate in the 350-775 nm range.

Co(II)–Versatic Acid 10

A two-stage deconvolution procedure was applied to the absorption spectrum of Ni(II)-loaded Versatic Acid 10. First, the charge transfer band was fitted (least squares) using a Voigt profile over two spectral ranges: 350–450 nm and 775–950 nm. This excludes the region in which d-d transitions overlap with the charge transfer band. The maximum was fixed at 358 nm. The resulting fit had an adjusted R^2 value of 0.99928, and served as a baseline for the analysis of the d-d transition bands.

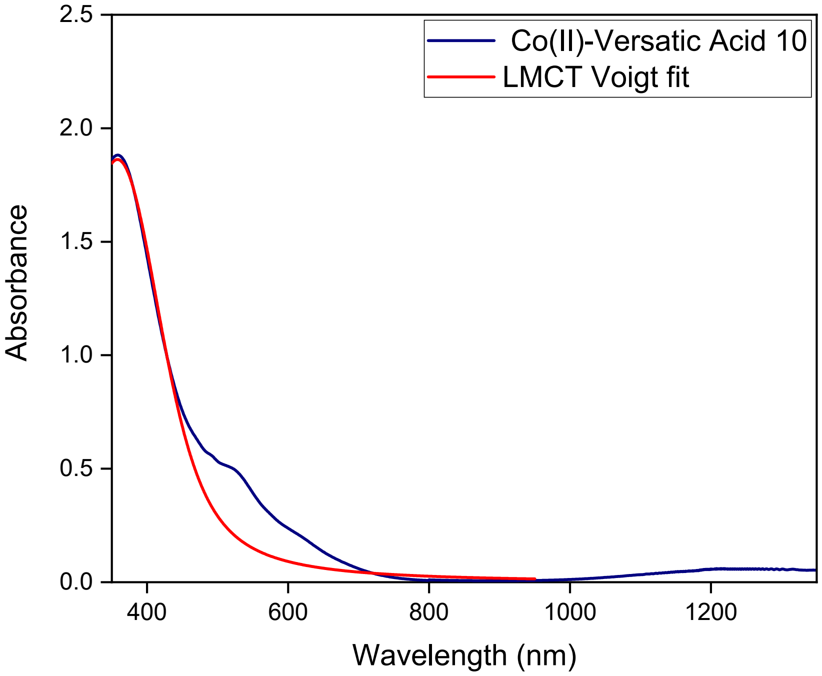

Fig. S4: Least square fit of a Voigt profile to the absorption spectrum of Co(II)-loaded Versatic Acid 10 in the 350-450 and 775-950 nm ranges.

The resulting function was subtracted from the original absorption spectrum, and a least-square fit of four Voigt profiles was applied to the result between 450 and 800 nm. The cumulative fit had an adjusted R^2 value of 0.99985, with maxima at 468, 491 and 522 nm for the components of the $^4T_{1g}(F) \rightarrow {}^4T_{1g}(P)$ manifold and at 577 nm for the $^4T_{1g}(F) \rightarrow {}^4A_{1g}(F)$ transition.

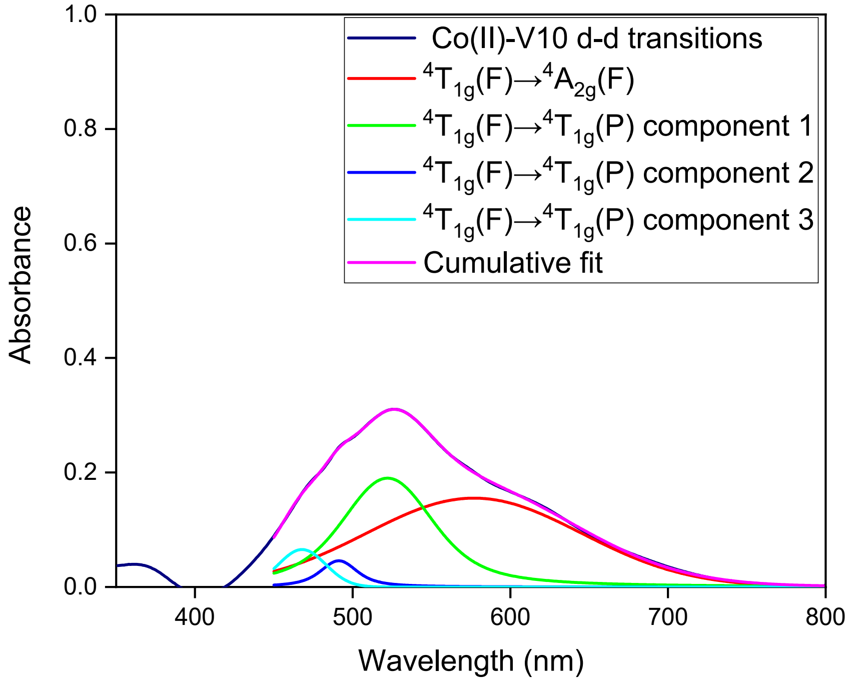

Fig. S5: Least square fit of four Voigt profiles to the absorption spectrum of Co(II)-loaded Versatic Acid 10 in the 450-800 nm range, after subtraction of the Voigt function fitted to the charge transfer band.

2. Absorption spectra of hydrated Ni(II)–loaded extractants

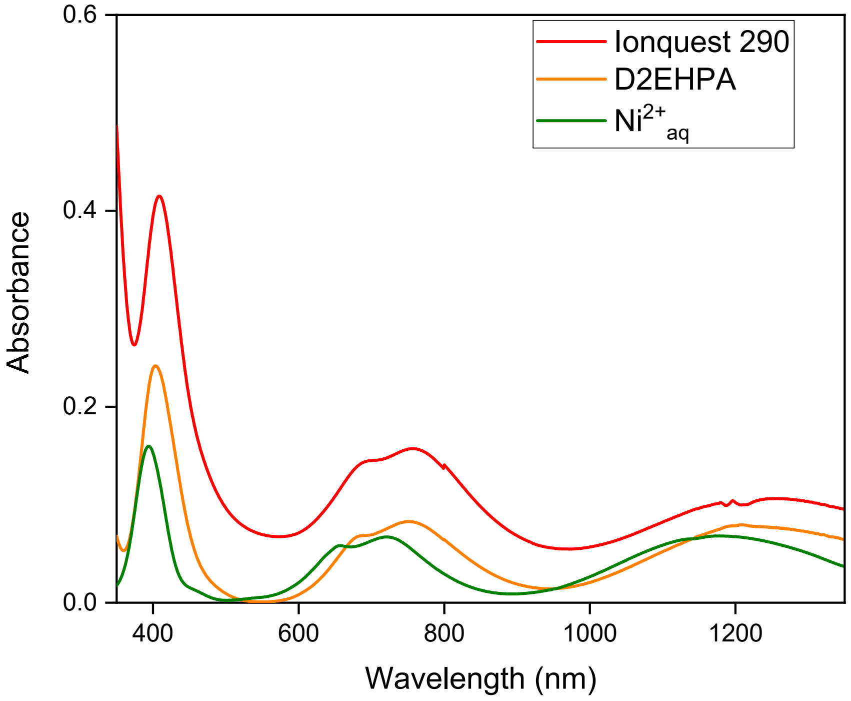

Fig. S6: UV-VIS-NIR absorption spectra of aqueous Ni(II) sulfate and hydrated samples of Ionquest 290 and D2EHPA loaded with Ni(II) (100% loading, 10wt% nickel-extractant salt in heptane). Spectra were normalized to a path length of 2 mm

4. LFSE calculation for Cu(II)-Mextral 54-100

As the barycenter of the d-orbital energies must remain unchanged irrespective of the coordination geometry, the energies of the d-orbitals must vary linearly with a parameter α which describes the deviation from an octahedral crystal field. A value of $\alpha = 0$ corresponds to perfect octahedral symmetry, while $\alpha = 1$ denotes a square planar geometry. Intermediate values indicate axial distortion of the octahedron.

The bands visible in the spectrum of the Cu(II)-Mextral 54-100 complex correspond to the $d_{z2} \rightarrow d_{x2-y2}$ and $d_{xy} \rightarrow d_{x2-y2}$ transitions. In an octahedral complex, the d_{z2} and d_{x2-y2} orbitals are positioned at $+0.6\Delta_O$ while d_{xy} has an energy of $-0.4\ \Delta_O$. In a square planar crystal field, d_{x2-y2} is approximately positioned at $+1.23\Delta_O$, d_{xy} at $+0.23\Delta_O$ and d_{z2} at $-0.43\Delta_O$ for most complexes.

Thus, the energies are (expressed in terms of Δ_O):

$$E(d_{x^2-y^2}) = 0.63\alpha + 0.6 \qquad \text{(Eq. S1)}$$

$$E(d_{xy}) = 0.63\alpha - 0.4 \qquad \text{(Eq. S2)}$$

$$E(d_{z^2}) = -1.03\alpha + 0.6 \qquad \text{(Eq. S3)}$$

The observed energies of the bands are 1.49×10^3 cm^{-1} for the $d_{xy} \rightarrow d_{x2-y2}$ transition and 1.88×10^3 cm^{-1} for the $d_{z2} \rightarrow d_{x2-y2}$ transition, with a ratio of 1.26. As the former has an energy corresponding to Δ_O, d_{z2} must thus be positioned at $1.26\Delta_O$. This leads to a value of $\alpha = 0.76$. For a d^9 Cu(II) complex, this leads to an LFSE of $E(d_{x2-y2}) = 1.08\Delta_O$.

4. Absorption spectra of aged Co(II)-loaded samples

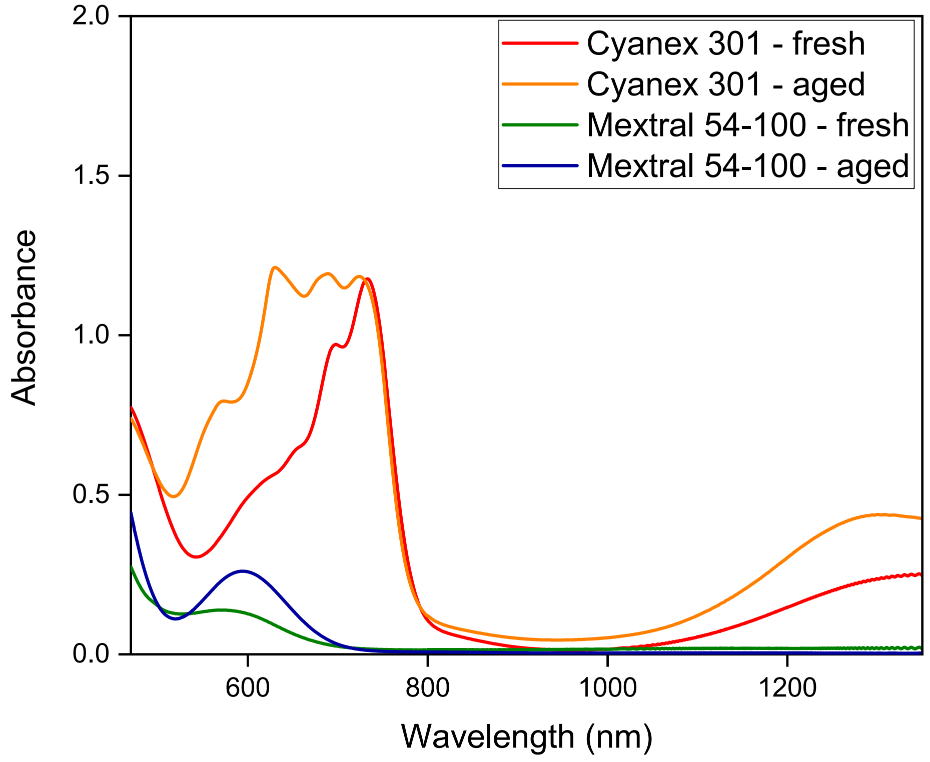

Fig.S7: UV-VIS-NIR absorption spectra of 0.1 mol L^{-1} solutions of extractant, at 20% loading with Co(II), measured one day vs. 10 days after equilibration. The Cyanex 301 samples were measured in a cell with 2 mm path length, while the Mextral 54-100 samples were normalized to a 1 mm path length..

5. Photographs of samples

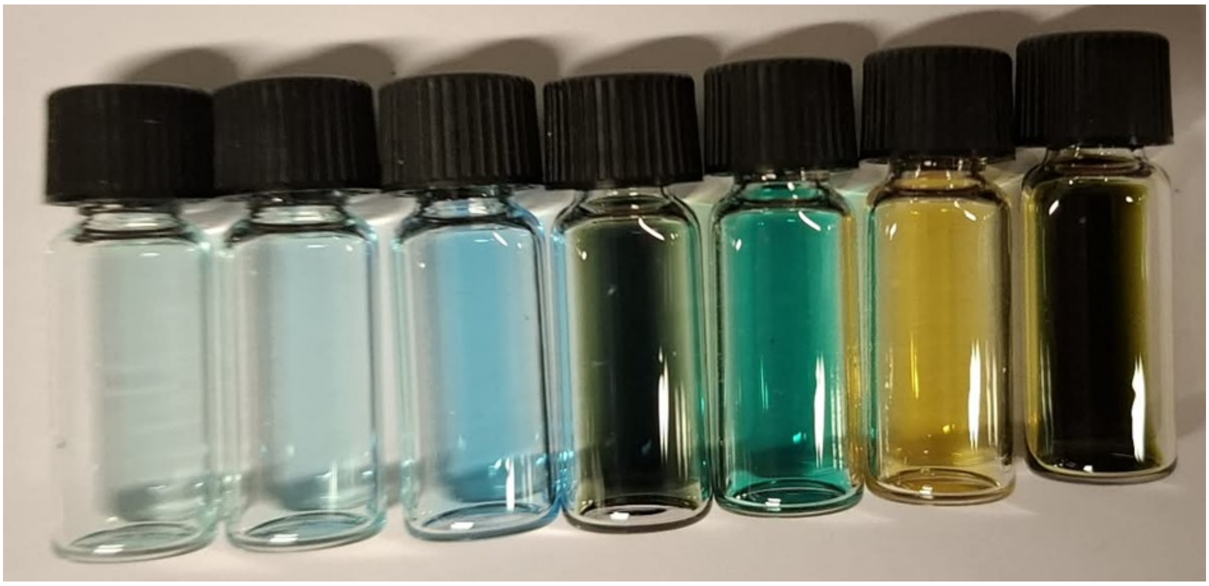

Fig. S8: Visual appearance of the Cu(II)-loaded samples (1 mol L^{-1} extractant in heptane, 20% loading). From left to right: D2EHPA, Mextral P507, Ionquest 290, Mextral 54-100, Versatic Acid 10, Cyanex 301, LIX 84-I.

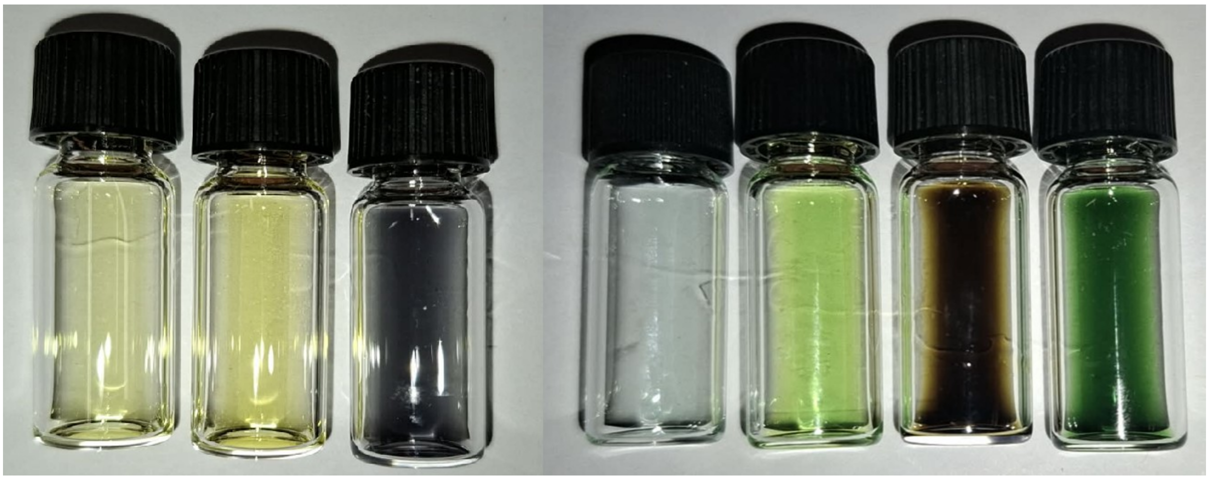

Fig. S9: Visual appearance of the Ni(II)-loaded samples (1 mol L^{-1} extractant in heptane, 20% loading). From left to right: D2EHPA, Mextral P507, D2EHPA, Versatic Acid 10,MExtral 54-100, Cyanex 301, LIX 84-I. The samples of D2EHPA, Mextral P507, Ionquest 290 were dried overnight over 3Å molecular sieves.

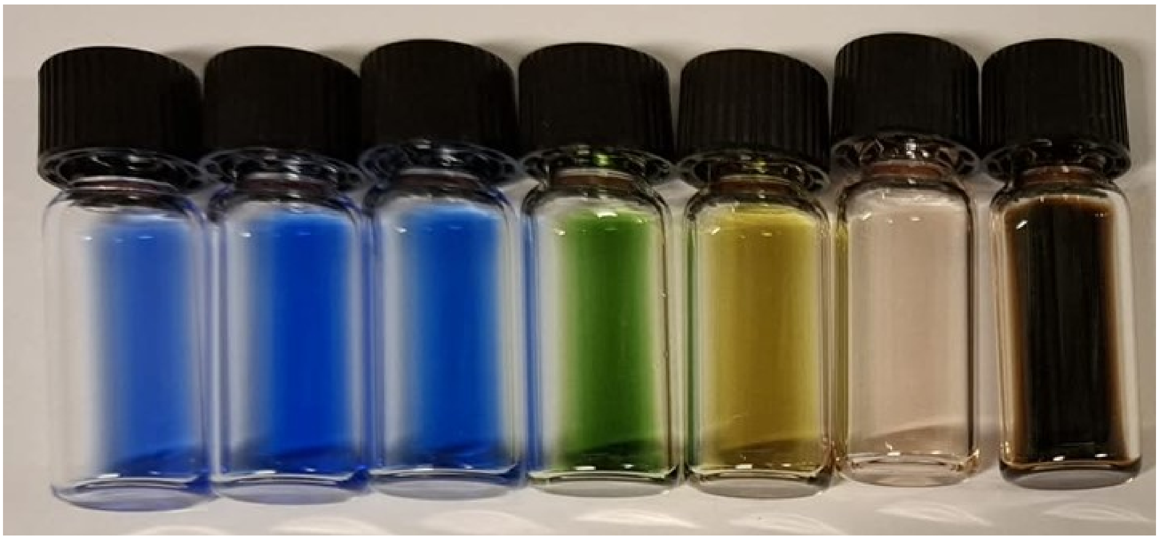

Fig. S10: Visual appearance of the Co(II)-loaded samples (1 mol L^{-1} extractant in heptane, 20% loading), taken the day after equilibration. From left to right: D2EHPA, Mextral P507, Ionquest 290, Cyanex 301, Mextral 54-100, Versatic Acid 10, LIX 84-I.

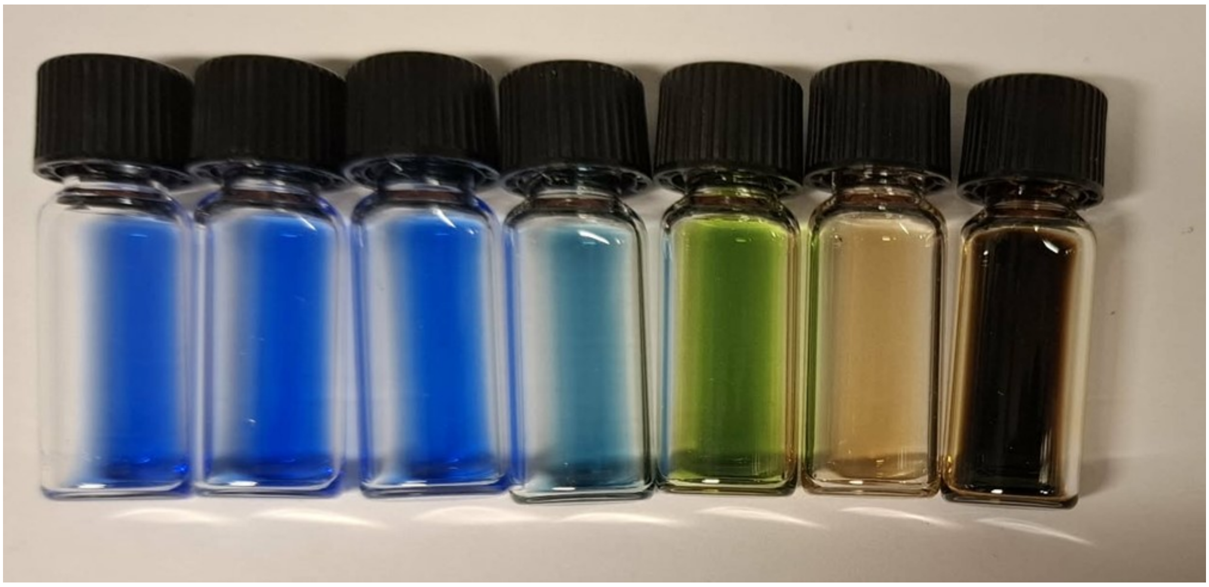

Fig. S11: Visual appearance of the Co(II)-loaded samples (1 mol L^{-1} extractant in heptane, 20% loading), taken 10 days after equilibration. From left to right: D2EHPA, Mextral P507, Ionquest 290, Cyanex 301, Mextral 54-100, Versatic Acid 10, LIX 84-I.
